# Nonclassicality Invariant of General Two-Mode Gaussian States

**DOI:** 10.1038/srep26523

**Published:** 2016-05-23

**Authors:** Ievgen I. Arkhipov, Jan Peřina Jr., Jiří Svozilík, Adam Miranowicz

**Affiliations:** 1 RCPTM, Joint Laboratory of Optics of Palacký University and Institute of Physics of the Czech Academy of Sciences, 17. listopadu 12, 77146 Olomouc, Czech Republic; 2 Faculty of Physics, Adam Mickiewicz University, PL-61-614 Poznan, Poland

## Abstract

We introduce a new quantity for describing nonclassicality of an arbitrary optical two-mode Gaussian state which remains invariant under any global photon-number preserving unitary transformation of the covariance matrix of the state. The invariant naturally splits into an entanglement monotone and local-nonclassicality quantifiers applied to the reduced states. This shows how entanglement can be converted into local squeezing and vice versa. Twin beams and their transformations at a beam splitter are analyzed as an example providing squeezed light. An extension of this approach to pure three-mode Gaussian states is given.

Despite of several decades of active research, the nonclassical properties of light remain one of the most intriguing problems in quantum optics (for a review see, e.g., refs 1, 2, 3, 4). A widely accepted criterion to distinguish nonclassical states from the classical ones says that a quantum state is nonclassical if its Glauber-Sudarshan *P* function fails to have the properties of a probability density^5^,^6^.

For practical purposes, several operational criteria for determining nonclassicality of either single-mode^7^,^8^,^9^,^10^,^11^ or multimode^9^,^11^,^12^,^13^,^14^,^15^ fields have been derived using the fields’ moments^8^,^12^,^14^,^16^ or the Bochner theorem^17^. Alternatively, the majorization theory also provides useful criteria^18^. Nonclassicality can directly be identified according to its definition when the quasidistributions of fields’ amplitudes^19^ or integrated intensities^20^ are reconstructed. The nonclassicality, which can be revealed in the continuous variables domain is becoming one of the most promising resourse for quantum communication technologies^21^.

Up to now the two most widely studied kinds of nonclassical light in the continuous variable domain are those exhibiting squeezing and entanglement. Both kinds of light have recently been recognized as potentially interesting not only for fundamental physical experiments but also for many applications in quantum technologies^21^,^22^,^23^,^24^,^25^. Both squeezed and entangled light can easily be generated in nonlinear processes, e.g., in second-subharmonic generation and parametric down-conversion, respectively.

In these processes, the optical fields are generated in Gaussian states. It has been shown in refs 26 and 27 that the Gaussian states obtained in both processes are mutually connected by linear transformations easily accessible by ‘passive’ linear optics. A suitable linear transformation then allows to obtain an entangled state at the expense of the original squeezed state under suitable conditions. Also, entanglement can serve as the source of squeezed light generated after suitable linear-optical transformations. Here, we explicitly reveal the conditions for the transformations of squeezed light into entangled light and vice versa by constructing a suitable global nonclassicality invariant (NI) that is composed of the additive identifiers of entanglement and local nonclassicalities (e.g. squeezing).

This allows rigorous control of the transformations of nonclassical resources (encompassing both local nonclassicalities and entanglement) in quantum-information protocols. Another example of importance of our result is the capability of testing the performance of schemes for the nonclassicality quantification based on transforming local nonclassicalities into entanglement^10^. Such schemes are considered as important as the determination of, e.g., the Lee nonclassicality depth^28^ or the Hillery nonclassical distance^29^, which are commonly used as nonclassicality measures, need the reconstruction of the *P* function. On the other hand, several measures of entanglement are known both for discrete and continuous quantum systems^11^,^23^,^30^,^31^,^32^,^33^,^34^. An intimate relation between entanglement and nonclassicality of, in general, noisy twin beams has recently been revealed in ref. 35. A general approach for analyzing this relation has been proposed in ref. 36 considering two-mode states. On the other hand, this NI allows to explicitly determine the entanglement of a given Gaussian state through local squeezing of the reduced single-mode states^37^.

From the general point of view, entanglement implies global nonclassicality of the overall field. On the other hand, nonclassical multimode fields do not necessarily have to be composed of mutually entangled parts. This occurs, when the parts as such exhibit marginal (local) nonclassicalities. Examples studied earlier have indicated that the action of global unitary transformations may be viewed as a ‘certain flow’ of entanglement into local nonclassicalities and vice versa. We note that, in the case of Gaussian fields, only the global unitary transformations, which preserve the overall number of photons, are naturally considered here. Such transformations are realized by passive optical devices and, from the mathematical point of view, they belong to the unitary group *U*(*n*). Indeed, there exists a tight relation between entanglement and local nonclassicalities which originates in the existence of a global nonclassicality invariant which splits into entanglement and local nonclassicalities quantifiers. In the past, an attempt to find such NI for single-mode Gaussian states and the vacuum was done in ref. 38 considering the logarithmic negativity^23^ as an entanglement measure and the Lee nonclassicality depth as a local nonclassicality measure. However, this approach worked only under quite specific conditions. On the other hand, the approach based on a global invariant succeeded when amplitude coherence and entanglement quantified by the maximal violation of the Bell-CHSH inequality have been analyzed together for a general two-qubit state^39^.

Here, considering two-mode Gaussian states, we reveal a nonclassicality invariant resistant against any passive (i.e., photon-number preserving) unitary transformation of their covariance matrix. We show that this invariant naturally decomposes into the expressions giving the local nonclassicality and entanglement quantifiers, which are monotones of the Lee nonclassicality depth and the logarithmic negativity, respectively. A global nonclassicality invariant is also suggested and verified for pure three-mode states.

In section *Theory*, general two-mode Gaussian states are analyzed. The generalization to pure three-mode Gaussian fields is given in section *Extension to pure three-mode Gaussian states*.

## Theory

The characteristic function or, equivalently, the corresponding complex covariance matrix **A**, can be used for the description of a Gaussian bipartite state with its statistical operator 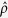 as follows:


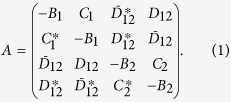


The normally-ordered characteristic function is then expressed as 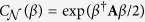 using the vector 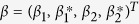. Elements of the covariance matrix **A** in Eq. (1) are defined as^40^


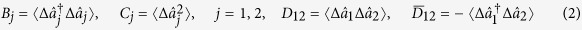


using the annihilation 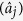 and creation 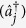 operators of mode *j*, *j* = 1, 2.

The negative determinants 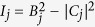 (*j* = 1, 2) of the diagonal blocks of the covariance matrix **A** immediately determine local nonclassicalities of modes 1 and 2. Indeed, the Fourier transform of the normal characteristic function of mode 1^2^ given as 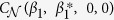 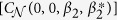 diverges if *I*_1_ < 0 [*I*_2_ < 0]. Determinant *I*_*j*_ is a monotone of the Lee nonclassicality depth *τ*_*j*_ of mode *j* that is given as the maximal eigenvalue of the *j*th diagonal block of the matrix **A**; i.e., *τ*_*j*_ = |*C*_*j*_| − *B*_*j*_^28^. Admitting also negative values for *τ*_*j*_ which can quantify the distance from the quantum-classical border we reveal the following monotonous relation:


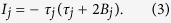


As the determinants *I*_*j*_ are invariant under local unitary transformations, we may define the local nonclassicality invariants (LNI) 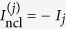, which quantify the local nonclassicalities.

On the other hand, the separability criterion for a two-mode state 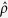 derived in refs 32, 41 and 42, which is based on the positive partial transposition (PPT) of 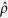, can be used to quantify the entanglement of 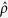 as


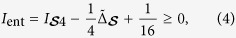


where 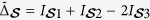. Equality in Eq. (4) holds for separable Gaussian fields. In Eq. (4), 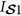, 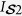, and 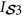 are the local invariants and 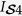 is a global invariant of the covariance matrix 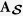 written for the symmetric ordering of field operators. As shown below, the quantity *I*_ent_, which we will call the entanglement invariant (EI), can serve as an entanglement quantifier since it is a monotone of the logarithmic negativity *E*_*N*_, i.e., it is also a monotone under unitary transformations^43^. The invariants 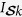 of the symmetrically-ordered covariance matrix 
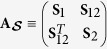
, as introduced in Eq. (4), are determined as 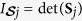, *j* = 1, 2, 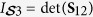, and 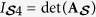.

The quantity 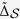 in Eq. (4), is related to the symplectic eigenvalue *d*_ of the partially transposed covariance matrix 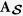 as follows^44^


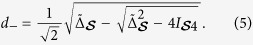


Combining Eqs. (4) and (5) we arrive at


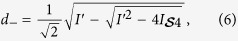


where 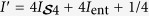. The eigenvalue *d*_ then gives the logarithmic negativity *E*_*N*_ as follows


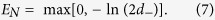


For pure states, we have 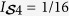 and the following monotonous relation between logarithmic negativity *E*_*N*_ and entanglement invariant *I*_ent_ can be given:


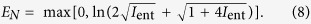


A detailed analysis of Eq. (6) confirms that, by keeping the global invariant 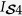 fixed, the EI *I*_ent_ remains a monotone of the logarithmic negativity *E*_*N*_ even for general two-mode Gausssian states.

It is easy to show that the global nonclassicality invariant (GNI) *I*_ncl_ defined as


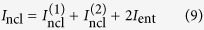


is invariant under any global passive unitary transformation applied simultaneously to both covariance matrices **A** and 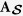. Using the definitions of 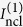, 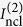, and *I*_ent_, together with the fact that the local invariant 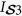 does not depend on operator ordering, we have


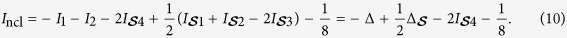


In Eq. (10), 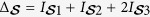 represents the global invariant of the symmetrically-ordered covariance matrix, whereas the quantity 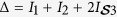 gives the global invariant of the normally-ordered covariance matrix.

For pure two-mode Gaussian states we have 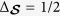, 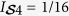, *I*_ncl_ = −Δ = *B*_1_ + *B*_2_, and 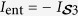. Therefore in this case, the GNI *I*_ncl_ is determined by invariants of the normally-ordered CM.

**Figure 1:**
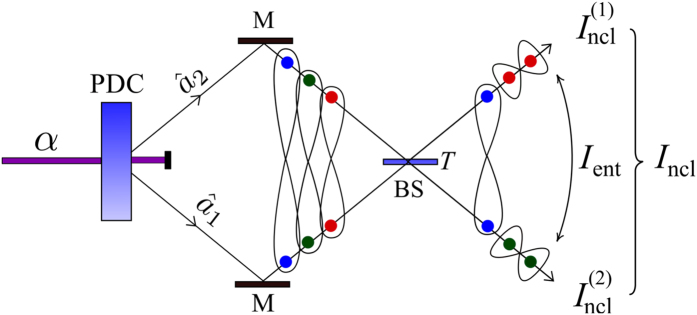
Pump field *α* generates photon pairs in the signal (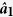) and idler (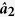) fields via parametric down-conversion (PDC). Photon pairs are mixed on a beam splitter (BS) with transmissivity *T*: photons in a pair either stick together (bunch) to contribute to squeezing or remain in different beam-splitter ports (antibunch) to form entanglement.

We note, that our invariant can also be applied to a single-mode Gaussian state. Specifically, this is a special case of our two-mode analysis if we assume that one of the input modes to the beam splitter (shown in Fig. 1) is in the vacuum state. This case is in analogy to the original approach of Asboth *et al.*^10^.

According to Eq. (9), which gives the central result of this paper, any passive unitary transformation modifies in general the LNIs 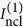 and 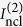 as well as the EI *I*_ent_, such that the value of the GNI *I*_ncl_ is unchanged. During such a transformation, the decrease (increase) of the local nonclassicalities has to be compensated by the increase (decrease) of entanglement. Thus, formula (9) represents a conservation law of the nonclassicality.

### Example: A twin beam (TWB) at a beam splitter

TWBs are provided by parametric down-conversion and, in their noiseless variant, are composed of many photon pairs with the twin photons embedded in the signal and idler fields. This guarantees strong entanglement in a TWB. As the marginal fields are thermal, no local nonclassicality is observed. Mixing of the signal and idler fields at the beam splitter represents a unitary transformation that modifies both entanglement and local nonclassicality as follows (for the setup, see Fig. 1).

The LNIs 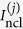 and EI *I*_ent_ acquire the form


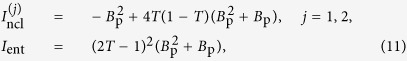


where *B*_p_ is the mean photon-pair number. According to Eq. (11), the LNIs 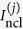 are given by two terms. The first (negative) term arises from the input thermal statistics and describes photon bunching. The second (positive) term is much more interesting as it describes the squeezing effect at a beam-splitter output port. At the ‘microscopic level’, this effect originates in pairing of photons in the output port caused by sticking of two twin photons at the beam splitter^3^,^26^,^45^. Such local pairing of photons creates local nonclassicalities of the field. The ‘sticking effect’ at the beam splitter reduces the number of photon pairs with photons found in different output ports and, so, it naturally reduces their entanglement, in agreement with Eq. (11). The strength of the relation between the micro- and macroscopic pictures is revealed when the formula for the GNI in Eq. (9) is written, *I*_ncl_ = 2*B*_p_. The GNI being linearly proportional to the number of photon pairs clearly shows that, in case of TWBs, only individual photon pairs are responsible for their entanglement and local nonclassicalities.

**Figure 2:**
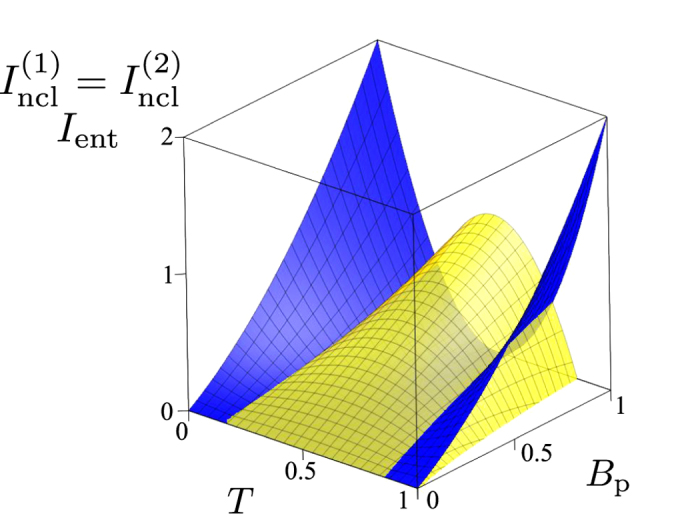
Local nonclassicality invariants 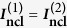 (yellow light surface) and entanglement invariant *I*_ent_ (blue dark surface) as functions of the mean photon-pair number *B*_p_ and transmissivity *T* for twin beams (only positive values are plotted).

Analyzing Eq. (11), the maxima in the LNIs 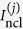 are reached for the balanced beam splitter (*T* = 1/2) that does not allow any entanglement^45^. The more unbalanced is the beam splitter, the greater is the *I*_ent_ and also the smaller are the LNIs 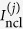. Local nonclassicalities of the output fields occur only for 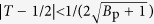. The quantification of this behavior is done in the graphs of Fig. 2 showing the LNIs 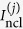 and EI *I*_ent_ as functions of the mean photon-pair number *B*_p_ and transmissivity *T*.

We note that, similarly as the input TWB may provide squeezed light at the beam-splitter outputs, the incident squeezed light present in one or both input ports allows for the generation of the entangled output fields.

## Extension to Pure Three-mode Gaussian States

Motivated by the results for two-mode Gaussian states, we suggest an appropriate form of a three-mode NI relying only on the LNIs and pairwise (two-mode) EIs. The proposed NI is invariant under any global passive unitary transformation provided that only pure three-mode Gaussian states are considered. This observation accords with the results in refs 26, 37 and 46 showing that (a) any entangled three-mode state can be transformed via a global unitary transformation into a state of three independent squeezed modes and (b) genuine three-mode entanglement can be expressed through the two-mode entanglements of three subsystems obtained by the reduction with respect to one mode. We note that this result applies also to the symmetric GHZ state in the continuous domain.

The symmetrically-ordered covariance matrix 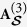 of a three-mode Gaussian state is written as


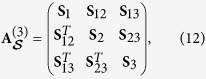


where the matrix **S**_*j*_ describes mode *j* and matrix **S**_*jk*_ characterizes the correlation between modes *j* and *k*. The matrices **S**_*jk*_ are independent of the operator ordering and, so, they occur also in the normally-ordered covariance matrix **A**^(3)^. We construct the three-mode GNI 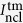 as follows


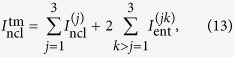


where 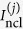 is the LNI of mode *j* and 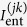 is the EI of modes *j* and *k* determined from their reduces statistical operator. Equation (13) can be rewritten as 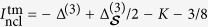, where 
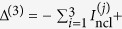
 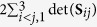, 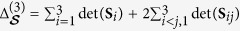, and 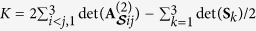 with 
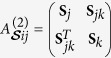
. Since 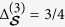 and 
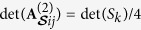
 for pure three-mode states, we have 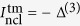. As Δ^(3)^ is a global invariant of the normally-ordered covariance matrix **A**^(3)^ under passive unitary transformations, the GNI 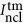 becomes unchanged when such transformations are applied. Similarly as for pure two-mode states, we have 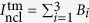, where *B*_*i*_ gives the mean number of photons in mode *i*. Therefore the GNI for pure three-mode state is determined by the local invariants of the normally-ordered covariance matrix **A**^(3)^. Formula (13) for the pure three-mode GNI 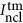 shows that the three-mode entanglement can be quantified by the sum of three two-mode entanglements. Monitoring the three LNIs and three EIs involved in Eq. (13) allows to quantitatively analyze the evolution of nonclassicality resources in any quantum-information protocol described by passive unitary transformations.

We note that the generalization to the case of *m* > 3 modes based on the assumption of two-mode entanglement quantifiers 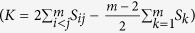 is not useful since the obtained quantity is not a global invariant, similarly as in the case of mixed three-mode states.

### Example: A twin beam transformed by two beam splitters

**Figure 3:**
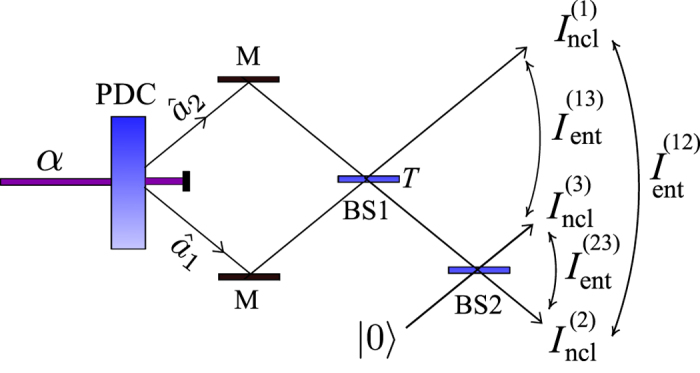
Pump field *α* generates photon pairs in the signal (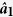) and idler (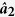) fields via parametric down-conversion (PDC). Photon pairs are mixed on a beam splitter (BS) with transmissivity *T*. Field in one output port of this beam splitter is combined with the vacuum |0〉 at another balanced beam splitter. LNIs 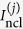 and EIs *I*_ent_(*jk*) characterized the three output fields.

A simple method providing varying bipartite entanglement among three output ports as well as locally nonclassical output fields can easily be constructed from the previous example of a TWB at a beam splitter. We enrich this method by additional splitting the field at the output port 2 by a balanced beam splitter with the output ports 2 and 3 (for the scheme, see Fig. 3)^47^,^48^.

This results in a general three-mode state. From the point of view of entanglement, photon pairs, which are originally responsible for the entanglement between modes 1 and 2, are divided by the second beam splitter to those establishing entanglement either in modes 1 and 2, or modes 1 and 3. On the other hand, the photon pairs, which are localized in mode 2 and responsible for its squeezing, may split at the second beam splitter giving rise to the entanglement between modes 2 and 3. This results in a full three-mode entanglement. Indeed, the presented theory provides the following formulas:


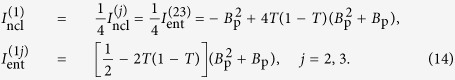


**Figure 4:**
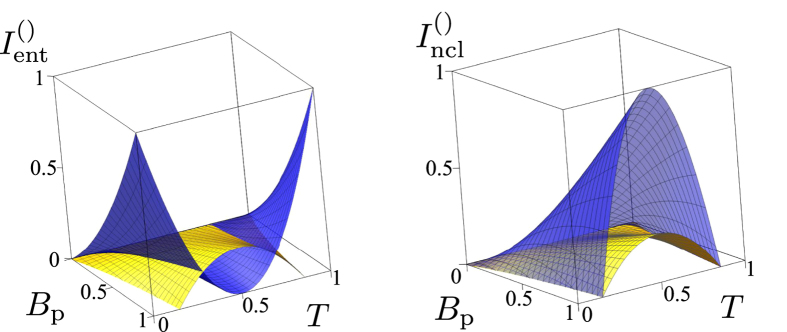
(**a**) Entanglement invariants 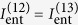 [blue dark surface] and 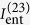 (yellow light surface) and (**b**) local nonclassicality invariants 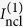 (blue dark surface) and 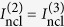 (yellow light surface) as they depend on the mean photon-pair number *B*_p_ and the beam-splitter transmissivity *T* for an initial pure TWB in the scheme of Fig. 3 (only positive values are shown).

These formulas are visualized in Fig. 4, which confirm our predictions. For the transmissivities *T* in certain interval found in the previous example and excluding *T* = 1/2, we have a genuine three-mode entanglement. Moreover all the three output fields are locally nonclassical. Whereas the LNIs 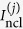 decrease with the increasing unbalance of the first beam splitter, the decrease of the EI 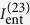 is compensated by the increase of the EIs 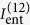 and 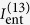. We note that the GNI is again linearly proportional to the initial photon-pair number *B*_p_, 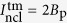.

### Critical analysis of the Asboth *et al.* scheme for nonclassicality quantification

If *T* = 1/2 in the above example, two separable squeezed states beyond the first beam splitter occur and, so, we retain the standard Asboth *et al.* approach^10^ for the nonclassicality quantification for the field in output port 2 of the first beam splitter. As certain amount of squeezed photon pairs remains in the output fields 2 and 3 beyond the second beam splitter, the standard approach cannot provide a full quantification of the nonclassicality of the analyzed field. Nevertheless, the EI 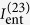 accessible in the Asboth *et al.* method provides a good estimate of the nonclassicality of the analyzed field since, according to Eq. (14), the LNI 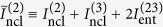 is linearly proportional to the EI 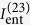 for an arbitrary transmissivity *T*.

## Conclusions

We have found an invariant for general two-mode Gaussian states which comprises the terms describing both marginal nonclassicalities of the reduced states and the entanglement of the whole system. Those terms being monotones under any unitary transformation of the Lee nonclassicality depth and the logarithmic negativity, respectively, quantify the flow of nonclasical resources when passive unitary transformations are applied. We gave the extension of these results to pure three-mode Gaussian states. As examples, we found a relation between twin beams and squeezed states. Moreover we critically analyzed the Asboth *et al.* method for quantifying nonclassicality.

## Additional Information

**How to cite this article**: Arkhipov, I. I. *et al.* Nonclassicality Invariant of General Two-Mode Gaussian States. *Sci. Rep.* **6**, 26523; doi: 10.1038/srep26523 (2016).
